# Advertisement of the Journal

**Published:** 1858-06

**Authors:** 


*Advertisement of the Journal.*—We desire to call attention to our advertisement, which appears on the second page of the cover of this number. The new management is prepared to issue the Journal in as good, if not a better form, than heretofore, and will devote to it all the attention which may be required. In order, however, that it may be handsomely sustained, it is necessary for our subscribers to remit promptly the amount of subscription; if thus remitted, $2.00 will be received, and on the prompt receipt from *new subscribers*, of the full amount, $2.50, we will send the *entire thirteenth volume*, of which we have a quantity on hand, so long as the copies last. An increased effort will be made to extend the circulation, and we would beg of our subscribers to aid us in this effort, as we contemplate, if encouraged, enlarging the Journal, a step which the amount of contribution to its pages will abundantly warrant, while we are only waiting for similar encouragement in its receipts.

The advertising department we would call especial attention to, from all who may feel it for their interest to advertise in any journal; and they should remember that this is the only one in Western New York, and that it comes to the eye of almost every physician practising in that section, besides many in nearly every state in the Union.

Prof. Hunt has retired entirely from the editorship and proprietorship, and has assumed all the back debts; we appeal to the good faith of our subscribers to make him whole, by sending the amount of their indebtedness as soon as they receive their bills; his labors in their behalf merits this consideration. All back remittances will be sent to his address, as we have nothing to do with them; all subscriptions for the fourteenth volume, however, may be sent to the publisher, E. R. Jewett.

